# Long COVID may not be explained by skeletal muscle involvement, but rather by other, more compelling pathophysiological concepts

**DOI:** 10.1055/s-0045-1809404

**Published:** 2025-12-15

**Authors:** Josef Finsterer, Fulvio Alexandre Scorza, Carla A. Scorza

**Affiliations:** 1 Neurology & Neurophysiology Center, Neurology Department, Vienna, Austria.; 2 Universidade Federal de São Paulo, Escola Paulista de Medicina, São Paulo SP, Brazil.

Dear Editor,

We were interested to read the article by Kouyoumdjian et al. 1 on neuromuscular transmission function and isolated muscle fiber conduction velocity (MFCV) using concentric needles and stimulation with a monopolar needle in 16 patients with a history of infection by severe acute respiratory syndrome coronavirus 2 (SARS-CoV-2) but without long coronavirus disease 2019 (COVID-19) syndrome (LCS), and 16 patients with a history of SARS-CoV-2 infection and LCS. Only one patient without LCS and only two patients with LCS had at least one abnormal jitter parameter. 1 No other parameter was abnormal, neither in the controls nor in the patients with and without LCS. 1 The authors 1 concluded that pathology of muscle fibers or neuromuscular junctions does not contribute to LCS. The study is noteworthy, but some points should be discussed.

The first problem is that the study does not report whether any of the included patients with a history of SARS-CoV-2 infection presented muscle symptoms or myositis, idiopathic inflammatory myopathy (IIM) or rhabdomyolysis during the infection. 1 Infection by SARS-CoV-2 is known to be complicated by rhabdomyolysis, myositis, or IIM. 2 3 4 The lack of differences in single-fiber electromyography (EMG) parameters between COVID-19 patients with and without LCS symptoms suggests that none of the 16 patients with LCS symptoms presented muscle involvement during the acute infection.

The second point is that myositis-associated or myositis-specific antibodies were not determined in any of the included patients. 1 Patients with previous SARS-CoV-2 infection are known to have elevated myositis antibodies in almost 2/3 of the cases, regardless of whether or not they presented muscle symptoms during the infection. 5 The absence of these antibodies would explain why no pathology of muscle fibers or neuromuscular junctions was detected.

The third point is that the interval between the interview and the neurophysiological examination was between 6 and 24 months. 1 An interval of 2 years is quite long, and several other conditions may have developed since the SARS-CoV-2 infection that could explain abnormal single-fiber EMG parameters. How was it ruled out that causes other than the previous SARS-CoV-2 infection or the LCS were responsible for the abnormal electrophysiological findings?

The fourth point is that the single-fiber recording was performed with concentric needle electrodes, but not with single-fiber EMG needle electrodes. Since the recording area of concentric needle electrodes is much larger than that of single-fiber needle electrodes, concentric needles do not always guarantee that the recordings are really made from a single muscle fiber and not from several of them.

The fifth point is that not all patients with a history of SARS-CoV-2 infection have had their infection confirmed by polymerase chain reaction (PCR). 1 Since the antigen test has lower sensitivity and specificity compared with the PCR test, patients in whom the infection was only diagnosed by an antigen test should have been excluded from the analysis.

The sixth point is that immunological complications other than myopathy or neurotransmission disease have not been considered as a cause of LCS. For example, it has been suggested that patients with SARS-CoV-2 infection develop a viral reservoir, meaning that the virus remains in the body and replicates, causing chronic infection. It is also suspected that SARS-CoV-2 infection acutely triggers autoimmunity in the body, which has also been observed with other viruses, such as the Epstein-Barr virus. It is also conceivable that LCS is due to the reactivation of other viruses that we already carry in our bodies. Another hypothesis to explain LCS is based on the assumption that patients with severe SARS-CoV-2 infection are especially affected by LCS in the acute phase due to tissue damage and dysfunction.

In conclusion, this interesting study 1 has limitations that affect the results and their interpretation. Addressing these limitations could strengthen the conclusions and support the message of the study. The clinical presentation of LCS may not be explained by skeletal muscle impairment, but rather by other, more compelling pathophysiological concepts.
